# Marc Richelle (1930–2021) and the Study of Temporal Regulation of Behaviour in Animals

**DOI:** 10.5334/pb.1095

**Published:** 2021-10-26

**Authors:** John H. Wearden

**Affiliations:** 1 School of Psychology, Keele University, Keele, ST5 5BG, UK

**Keywords:** operant conditioning, fixed-interval, DRL, cross-species comparisons, psychopharmacology

## Abstract

This article discusses the contribution of Marc Richelle to the study of temporal regulation of behaviour in animals. Richelle was a pioneer of behavioural pharmacology in Europe in the 1960s, and some of his early pharmacological experiments, particular those involving chlordiazepoxide, are discussed. Richelle frequently tested drug effects on performance on fixed-interval (FI) and differential reinforcement of low rate (DRL) schedules. Much of his later work, conducted with Helga Lejeune, involved cross-species comparisons of performance on FI and DRL, and often focused on potential differences between “timing competence” and “timing performance”. His work provided an unrivalled body of research on operant behaviour in different species, involving research on animals as different as cats and fish. Much of the work was reviewed in Richelle and Lejeune’s 1980 book Time in Animal Behaviour, which contained particularly influential accounts of collateral behaviour and inter-species comparisons.

The late Marc Richelle made many important contributions to Psychology, some of which are highlighted in a commemorative volume edited by Lejeune, Macar, and Pouthas (1995). The present article is intended to provide a concise non-technical account of just one of these, his work on the *temporal regulation of behaviour* in animals. Richelle generally preferred the term *temporal regulation* to that of *timing* and, as will be seen later, some of his research suggests that this distinction is important. Temporal regulation refers to the fact that when animals are exposed to experimental arrangements involving temporal periodicities, or time requirements for reinforcement, their behaviour often adjusts so that its distribution in time adapts to the temporal features of the experimental situation. The term *temporal regulation* is thus a description of behaviour, rather than describing an internal process which *explains* behaviour. This temporal regulation may or may not be produced by underlying timing processes, and situations can be found, in Richelle’s own work, in which temporal regulation of behaviour and underlying timing processes can be dissociated, as will be seen later. Richelle’s position is reminiscent of that of De Houwer (2011) who argues for a distinction between *functional* and *cognitive* approaches to behaviour. In the present case *temporal regulation* would refer to a functional relation between behaviour and environmental manipulations, and *timing* to an underlying cognitive process.

In the present article I will try to provide some background about what Richelle did, as well as trying to show why it was important and original. I will also focus, although not completely exclusively, on publications where Richelle himself was an author or co-author, rather than reviewing the whole body of work on animal timing that has come from the laboratory he founded in Liège. But first, for non-specialists, I provide a description of two of the main techniques that he used in his research.

## Preamble: two schedules of reinforcement

In operant conditioning, schedules of reinforcement are experimenter-specified rules linking the emission of operant responses, such as key pecks or lever presses, to the delivery of reinforcers such as food pellets or grain. Schedules were developed in the pioneering work of Skinner (1938, see also Ferster & Skinner, 1957), and there are a vast number of them, but two were used very frequently in Richelle’s work. One of these was the *fixed-interval* (FI) schedule. The first response of the session is reinforced, and this response starts a clock. The clock runs for a fixed period of time (such as 60 s or 3 minutes), which is the schedule parameter, but nothing signals this elapsing time to the animal. When the clock times out, the next operant response is reinforced, and this restarts the clock, and the next interval, and so on. In FI, there is no requirement that any particular pattern of responses be emitted but, after a few hours of exposure, animals of most species develop what Richelle (1972) called *spontaneous temporal regulation* of behaviour. This usually takes the form of an accelerating rate of responding as the interval elapses, often with the response period being preceded by a substantial pause after reinforcement. Temporal regulation on a schedule like this can be precise, with most responses concentrated towards the time when the reinforcer is available, or less precise, with responses distributed more evenly as time in the interval passes. An index of the quality of temporal control used in many of Richelle’s articles is *the index of curvature* of Fry, Kelleher, and Cook (1960). This is calculated by dividing the time in the interval into a number of bins of equal length, usually 8, and calculating how steeply responding increases over the bins. High curvature values indicate more precise temporal regulation than lower values.

The other schedule that Richelle used in many studies is *differential reinforcement of low rate* (DRL). In spite of its complicated name the DRL procedure is very simple. In order to obtain a reinforcer a response must be spaced by *t* seconds or more from the previous response (where *t* is the schedule parameter). If the spacing is less than *t* the time requirement is reset from zero. Any temporal regulation which occurs on this schedule is not spontaneous, as the schedule itself imposes a time requirement for delivery of the reinforcer. There are different ways to measure performance on DRL. One is simply some measure of response efficiency, for example, the ratio of reinforced responses to non-reinforced ones. Another measure is a distributional plot of the times between responses (usually called *inter-response times*, or IRTs), where the frequency of IRTs of different durations is plotted against IRT length. So, for example, efficient performance on DRL would be manifested by a frequency distribution of responses centred around the DRL requirement; inefficient performance would involve a distribution with many unreinforced, and thus too short, IRTs.

In the case of both schedules of reinforcement the usual operant method, and one followed in Richelle’s work, would involve the animal being exposed to the schedule for a number of experimental sessions, often lasting an hour or more. The focus of interest is usually on “steady-state behaviour”, that is, the performance achieved after a number of hours of exposure to the schedule, often 20 hours or more, rather than the initial period of learning the schedule requirement.

## The early years: mostly psychopharmacology

A considerable number of Richelle’s early publications used operant methods to explore the effect of various drugs. According to Richelle himself (1991) his entry into psychopharmacology was in part almost accidental, as “chance comes into the picture” (p. 415) in determining that he would follow this research area. After his return from Harvard in 1959, Richelle intended to set up an animal operant conditioning laboratory in Liège, but there was no space in the Psychology Department for this. However, facilities were made available to him in the Liège Department of Pharmacology. As Richelle (1991, p. 416) puts it “Being a guest in a medical school laboratory, courtesy dictates that I would give something in return to my hosts … [so] it seemed obvious that I should start some drug experiments”. He had previously been introduced to operant behavioural psychopharmacology at Harvard by P.B. Dews and W.H. Morse, so was familiar with techniques in this area, and he embarked on a series of studies of the effects of various drugs on operant performance, usually involving FI or DRL schedules, from the early 1960s to what was probably his last publication on this topic, Lejeune et al. (1995). The unique circumstances of experiences at Harvard and establishment of a laboratory in a unit mostly devoted to pharmacology, however fortuitous they might have been, established Richelle as the continental European pioneer of behavioural pharmacology. For a history of his involvement, see Richelle (1991).

A complete review of all the psychopharmacological studies with which Richelle was involved is beyond the scope of this article and some of his early studies with cats produced data that were difficult to simply summarize or interpret, for example, different effects on different individual animals (Faidherbe, Schlag, & Richelle, 1961), problems with motivation to consume the reinforcer (Faidherbe, Richelle, & Schlag, 1962). However, many consistent and clear results were found, in particular with chlordiazepoxide. A typical early experiment was Richelle, Xhenseval, Fontaine, and Thone (1962). Here, rats were trained either on an FI 2 minute, or DRL 34 s, schedule, and different doses of chlordiazepoxide were administered. Increasing the drug dose increased response rate on both schedules, and in both cases also disrupted temporal control. That is, on FI the distribution of responses during the interval became flatter, and on DRL performance became more inefficient.

In an early review article, Richelle (1963) discusses the general contributions of experimental Psychology to psychopharmacology, in particular advocating the use of operant methods. In this article, he illustrates the effects of chlordiazepoxide in both a cat and a rat, who exhibit similar performance on a FI schedule, and a similar response-increasing effect of the drug. In the article he cites several advantages of the operant method, such as the fact that responses are recorded automatically, that stable behavioural baselines can be developed, and that each animal can be used as its own control, obviating the need for statistical analyses.

Consistent with the aim of “prediction and control of behaviour”, associated with Skinnerian behaviourism, usually no attempt was made in Richelle’s early pharmacological articles to explain the changes in behaviour resulting from drug administration in terms of underlying processes. For example, questions like whether the drugs disrupted sensitivity to time in some way, or whether they only altered response output or motivation, were not addressed. Only much later in Richelle’s work, as in Lejeune et al. (1995), for example, were issues like this explored. To illustrate, Lejeune et al. used a tricyclic antidepressant (amineptine) with rats on a DRL 30 s or FI 60 s schedule. Like chlordiazepoxide, this drug increased response output and worsened temporal control (as illustrated on FI by a decrease in the index of curvature, for example). However, a second experiment used a bisection procedure. Here, to simplify slightly, two operant levers were used, and a response to one of them was reinforced after a 2 s auditory stimulus and a response to the other after an 8 s stimulus. When this time discrimination had been established the drug was administered, but the drug had no effect on performance accuracy, although response latencies were changed. The conclusion drawn by Lejeune et al. (1995) was that timing capacity had not been affected by the drug, although timing performance certainly was, and it is possible that this was also the case in some of Richelle’s earlier studies.

If Richelle was, at least in part, an accidental psychopharmacologist, he was certainly an active one, producing more than twenty articles on the subject, and work on psychopharmacology and behavioural neuroscience continues to this day in the laboratory he founded (e.g., Didone, van Ingelgom, Tirelli, & Quartemont, 2019; Serrano et al., 2019). However, as well as pharmacological work, in his early years Richelle also conducted some purely behavioural studies, and I will mention two of the more unusual ones.

In the Department of Pharmacology in Liège in the early 1960s some physiological research was being carried out on cats. These are a very unusual experimental species for operant research but common at the time in neurophysiological studies. Cats were used in some drug experiments (e.g., Faidherbe et al., 1961) but Richelle was also involved in some purely behavioural studies. One of these (Richelle, 1965) describes the development of an “experimental neurosis” in cats. This term had previously been used to describe results in some experiments by Pavlov (1927), and the “neurosis” involved not only disruption to the behaviour learned on the experimental task, but also changes in an animal’s behaviour outside the laboratory. In Pavlov’s experiments, these “neuroses” often arose when two different but very similar conditioned stimuli were contrasted, one followed by food and the other not. The difficulty of the discrimination could result in an “experimental neurosis” in some subjects. Mineka and Kihlstrom (1978) provide a general review of conditions under which such “experimental neuroses” occur.

Richelle (1965 and 1972) reported “experimental neuroses” produced in cats by procedures involving simple food reinforcement. For example, in Richelle (1965) cats were transferred from their well-learned FI 2 minute schedule to another (fixed-ratio) were they were required to make a fixed number of responses for each reinforcer delivery. That is, the schedule requirement was changed from one involving time to one involving number of responses. This change produced a dramatic deterioration in operant responding, as well as effects on the animal’s behaviour outside the apparatus. In contrast, naïve animals would perform on fixed-ratio schedules without any difficulty. Likewise, Richelle (1972) reports data from Macar (1971) showing that exposure of cats to lengthy periods of DRL resulted in a gradual reduction to nearly zero in their operant responding.

Another rather unusual early purely behavioural experiment used hamsters. These animals will respond for food rewards, but prefer to hoard them rather than eating them immediately. Richelle et al. (1967) developed an apparatus in which a Skinner box was attached to another chamber in which the animals could hoard their food pellets. Removing the hoarded pellets substantially increased response rate compared with the situation where the pellets were left untouched, suggesting that the hoarding of the pellets was itself reinforcing. This provided an early example of a relation between performance on an operant conditioning schedule and a natural behaviour of the species used, something which later became emphasized in the “constraints on learning” literature (e.g., Shettleworth, 1972), discussed later in this article.

We see in publications from the 1960s some trends that were to mark Richelle’s later work on animal timing of a purely behavioural sort. One of these was the consistent use of different schedules that were time-related, particularly FI and DRL. The other trend was the use of different, and sometimes unusual, animal species.

## “Time in Animal Behaviour”: before and after

The high point of Richelle’s technical research on animal timing was almost certainly the publication of *Time in Animal Behaviour* (Richelle & Lejeune, 1980). However, some important issues were prefigured in earlier work. In particular, a book chapter by Richelle (1972), which drew extensively on publications by Lejeune (1971) and Macar (1971), introduced an important theme of Richelle’s studies on animal timing more generally, which he later (Richelle & Lejeune, 1984) called “timing competence” as opposed to “timing performance”.

Richelle (1972) reported some striking, and apparently paradoxical, differences between performance on FI and DRL schedules in a number of species. For example, on FI schedules of up to 8 minutes, cats would usually pause for long periods of time after reinforcement, sometimes for 5 minutes, before resuming responding. However, on a DRL schedule they were unable to space their responses by more than 1 or 2 minutes. As Richelle (1972, p. 233) puts it “Why is the animal able to pause spontaneously for 5 min or so when it is not required and *not* able to pause for 120 sec when this is the condition for reinforcement?” (his italics). This problem was not restricted to cats, and Richelle reported data from a thesis by Mantanus carried out in the Liège laboratory, where mice were able to produce normal temporal regulation on FI schedules up to 4 minutes long, but unable to adjust well to DRL 30 s. Pigeons, likewise, could exhibit long pauses on FI, but adjusted very poorly to shorter DRL values, often emitting large numbers of rapidly- produced responses even though the DRL schedule required spaced responding. In the 1972 chapter, Richelle offered the tentative proposal that some sort of species difference in ability to inhibit responses might be the cause. I will return to this issue later.

*Time in Animal Behavior* (Richelle & Lejeune, 1980) had several contributors (Daniel Defays, Pamela Greenwood, Francoise Macar, and Huguette Mantanus) besides the two cited authors, although they were responsible for the majority of the text. The book covers almost everything known about animal timing up to the date of its publication. It is a work of almost unsurpassed thoroughness, and has no more modern equivalent for anyone interested in learning about the topic of its title. The main difference from something written today would be that a modern text would probably include substantial sections on theories related to animal timing, in particular *Scalar Expectancy Theory* (*SET*, see Gibbon, Church, & Meck, 1984) although, of course, this was not developed until after *Time in Animal Behaviour* had been published. I discuss here two chapters which contain material that was subsequently influential.

The chapter on *Collateral Behaviour*, in spite of being very short, was one of the chapters most frequently cited by later authors. Collateral behaviours are those that animals produce on schedules, often on those involving temporal regulation, but which are usually not measured by the experimenter. For example, on a DRL schedule, an animal might emit a regular sequence of activities, such as movements around the operant chamber, grooming, and so on, between the measured operant responses. These are *collateral behaviours*, and Richelle and Lejeune (1980) reviewed early work which not only found such collateral behaviours on a range of temporally-regulated schedules, and in different species, but also raised the important questions of whether these collateral behaviour (a) aided temporal regulation of the measured responses and (b) were *necessary* for good temporal regulation of behaviour.

They found instances were collateral behaviours appeared to aid temporal regulation, but question of whether they were actually necessary became particularly relevant with the development of the *Behavioral Theory of Timing* (*BeT*) by Killeen and Fetterman (1988). Lejeune, Richelle, and Wearden (2006) discuss this theory in some depth, in what was probably Richelle’s last publication on animal timing, and that article could be consulted for more details. However, the basic idea of *BeT* is very simple: on a schedule like FI, *BeT* proposes that the animal does not “time” the interval at all, but instead produces a stereotyped sequence of collateral behaviours, which are usually not observed by the experimenter. One of these collateral behaviours at the end of this behavioural chain acts as a cue for the operant response, which is then produced, and measured. Similarly, a sequence of collateral behaviours might mediate performance on a DRL schedule, if a consistent sequence of such behaviours occurs between the measured operant responses. As Lejeune et al. (2006) point out, the main intellectual motivation for theories like *BeT* was a radical-behaviourist desire to explain temporal regulation in animals without postulating underlying processes such as “internal clocks” that an animal can read, as used by *SET*. To return to Richelle’s preference for the term temporal regulation as opposed to timing, *BeT* attempts to account for temporal regulation *without* underlying timing processes.

The original exposition of *BeT*, and other articles sympathetic to this theory, often cite Richelle and Lejeune’s review of collateral behaviour in *Time in Animal Behaviour* as if it supported, at least implicitly, the view that “behavior is a mediator of temporal control” (in the words of Killeen & Fetterman, 1988, p. 274). In fact, Richelle and Lejeune’s conclusion was the opposite. In their own words (p. 198) “collateral behaviours do not, in any strict sense, *mediate* time estimation. They play at best an auxiliary function in temporal regulation”. For a more detailed discussion of the possible role of collateral behaviours, another publication from the Liège laboratory, albeit not one with Richelle as an author, is Lejeune, Ferreira, Cornet, and Wearden (1998).

The other chapter in *Time in Animal Behaviour* which influenced later work was that which discussed species differences in the temporal regulation of behaviour, and apparent difficulties that some animal species had in adjusting their behaviour to the requirements of spaced-responding schedules like DRL. These issues were both relevant to a popular topic in the animal Psychology of the 1970s and 1980s, that of “constraints on learning” (for a review, see Shettleworth, 1972). It was at one time believed that standard animal learning techniques such as Pavlovian and operant conditioning could be used to manipulate *any* response in animals, given appropriate motivation and provision of an appropriate reinforcer. The discovery of phenomena such a taste-aversion learning (Garcia & Koelling, 1966) suggested, instead, that animal learning depended, at least in part, on the biological propensities of the animal studied, so not all responses were equally “conditionable”. In an early article (Richelle, 1963) Richelle seemed to subscribe to the view, common in the early years of operant research (e.g., Skinner, 1950), that the performance of different animal species on schedules of reinforcement would be expected to be similar. However, by the 1970s, his position had clearly changed towards one of exploring cross-species similarities and differences in operant behaviour.

*Time in Animal Behavior* contains much material on operant behaviour in different animal species (e.g., in its chapter 4), but perhaps the clearest and most succinct exposition of Richelle’s position on the interpretation of potential species differences in behaviour comes in Richelle and Lejeune (1984). The authors discuss three different “hypotheses” about species differences in temporal regulation. One is an *evolutionary hypothesis* “Temporal regulations of behaviour become more refined and efficient as we climb the phyletic scale”. Another is the *egalitarian hypothesis* “The capacity for temporal regulation is equally distributed among all species”, although demonstrating this may depend on an appropriate choice of response and reinforcer, for example. Finally, there is an *ecological hypothesis* “Different species would exhibit different capacities for temporal regulation as a function of the particular repertoire evolved under selective pressure” (all quotes from Richelle & Lejeune, 1984, p. 255).

Richelle and Lejeune (1984) reprised some material from *Time in Animal Behavior*, but added data from some new studies, usually from the Liège laboratory. As mentioned earlier, the Fry et al. index of curvature under FI can be used to measure the quality of temporal regulation on this schedule with higher values indicating better regulation. Comparison of different species on this measure reveals many differences. At first, there seems to be support for the evolutionary hypothesis, as mammals like cats, rats and mice exhibit the highest values. However, not all mammals show good temporal regulation, for example, the *potto edwarsis*, a type of lemur, performs poorly. After mammals come birds, with fish and turtles the worst performers. A comparison of DRL performance, using median IRT, reveals a similar, if slightly more complicated, picture. Cats and rats can space their responses well on this schedule, at least up to values of 20 s or so, whereas pigeons are very poor, as were turtledoves. However, the *potto edwarsis* performed well on DRL, even when the response spacing requirement was up to 60 s, in contrast to its poor performance on FI. Laboratory mice, as opposed to cats and rats, performed poorly or DRL, but wild mice (woodmice) showed much better performance on this schedule than their laboratory counterparts.

The case of the wild and laboratory mice shows that not all closely-related animal species behave in the same way, and another example of this from Richelle’s work comes from a comparison of pigeons with turtledoves. Lejeune and Richelle (1982) used FI schedules of from 2 to 10 minutes long with rats, pigeons, and turtledoves. Temporal regulation of behaviour was most precise in the rats, and poorer for pigeons and doves. However, perhaps the most striking finding is that of marked differences in performance between the pigeons and doves, the latter species producing much lower curvature indices, and much more even distribution of behaviour within the interval, indicating poorer temporal regulation.

Richelle and Lejeune (1984) also discuss the question of the response used to assess temporal regulation. The key-peck in pigeons is a response that the animals make to stimuli associated with food, even if it is not required (the phenomenon of *autoshaping*, Brown & Jenkins, 1968), so may be difficult to inhibit in situations where food delivery is contingent on withholding key-pecks, such as DRL. This is in line with the suggestion of Richelle (1972) that problems involved in the inhibition of responses may be a reason for poor DRL performance in pigeons. This argument suggests that changing the response might reveal unsuspected performance ability, and this turns out to be true. Richelle and Lejeune report a contrast between DRL key-pecking in pigeons and a situation in which pigeons were required to remain on a perch for a period of time in a DRL-like contingency. Performance was vastly superior with the perching response. Lejeune & Jasslette (1986) later compared perching with the much more awkward response of treadle-pressing in pigeons, and found better performance with the more ethologically-appropriate perching. This dissociation between performance with different responses may justify Richelle’s usual insistence on the term *temporal regulation* rather than *timing*. His own work, for example, suggests that temporal regulation of pigeons’ pecking on DRL might be poor even though their underlying timing, as revealed when the response is changed, is much better than performance with key-pecks suggested.

In terms of the “hypotheses” advanced in the Richelle and Lejeune (1984) article, the results reviewed produced partial support for all three. It seems clear that position on the phyletic scale plays an important role in determining the quality of temporal regulation of behaviour, as the performance of mammals generally exhibits the best temporal regulation (hypothesis 1). On the other hand, the effects of changes in the response, for example in pigeons, shows that timing capacity can be masked by choice of an inappropriate behavioural measure, and that different animals may be more similar in terms of timing capacity than it appears at first sight (hypothesis 2), with the differences being related in some cases to ethological predispositions of the animals tested (hypothesis 3). The “hypotheses” do not, of course, account for all the effects obtained in the comparative studies in which Richelle was involved: one notable problem is why closely-related species (pigeons and turtledoves, woodmice and laboratory mice) sometimes behave very differently.

The work on comparison of the behaviour of different animal species conducted by Richelle and his associates shows just how complex questions about species differences in temporal regulation can be. Some intriguing puzzles, like the behavioural differences between pigeons and turtledoves, and wild and laboratory mice, have still not received any really definite solution. However, in spite of this, data on the performance of different animal species collected in the Liège laboratory, some of it derived from material presented in *Time in Animal Behaviour*, has been used to address some theoretical issues in animal timing. One of these was the question of whether the behaviour of different animal species conformed to the *scalar property* of time required by SET (Gibbon et al., 1984). The scalar property is the idea that the standard deviation of measures of timing behaviour should be a constant fraction of the mean measure as the interval timed changes, which generates a kind of proportional relation between responding and elapsed time. There are different ways of testing this, but on an FI schedule, the rate of responding at some fraction of the interval should be the same proportion of the rate at the end of the interval, whatever the interval length. For example, if the response rate at the end of an FI 30 s interval is x responses/second, and the rate at 15 s into the interval is y, the ratio y/x should be the same when the response rate half-way through an FI interval is measured, whatever the FI value is. Lejeune and Wearden (1991) showed that this was true over a large range of FI values for many animal species. That article also discusses a number of problematical theoretical issues involved in cross-species comparisons, not the least of which is that different species compared are hardly ever related in any direct evolutionary line.

It will not have escaped the reader’s notice that the vast majority of Richelle’s later publications reporting behavioural results have Helga Lejeune as a co-author, and no account of Richelle’s work on temporal regulation should omit her particular contribution, which cannot be overestimated. Lejeune was not only what Richelle (2006) called his “irreplaceable collaborator“, but she also published extensively, independently of Richelle, on both animal and human timing (see for example, the important and frequently-cited theoretical article, Lejeune, 1998).

## Conclusion

The large body of work on temporal regulation of behaviour in animals from the Liège laboratory, with which Richelle was actively involved for most of his career up until his retirement, perhaps contributed more to the study of cross-species comparisons in operant behaviour than research from any other laboratory in the world. Only a portion of the studies in which Richelle was involved, to say nothing of work which came later from the same laboratory, has been mentioned here. Richelle was indeed fortunate in the excellent quality of his students and collaborators, many of whom developed distinguished careers in diverse areas of Psychology in their own right. Nevertheless, he deserves credit for giving the initial impetus to this research in the late 1950s, and he nurtured and encouraged it for more than the 30 years of his career that followed. Temporal regulation of behaviour was, of course, only one of his many psychological interests (Lejeune et al., 1995), but it could be argued that his extensive and original work in this field was Marc Richelle’s most significant contribution to experimental Psychology.
